# Delayed traumatic pseudoaneurysm of a branch of the superior medial genicular artery treated with ultrasound-guided thrombin injection

**DOI:** 10.12701/jyms.2026.43.30

**Published:** 2026-04-14

**Authors:** Young-Nam Roh, Choshin Kim

**Affiliations:** Division of Transplantation and Vascular Surgery, Department of Surgery, Yeungnam University College of Medicine, Daegu, Korea

A 59-year-old woman with no significant medical history presented to the emergency department after being struck by a reversing delivery truck. She had sustained a right eyelid laceration, blowout fracture, multiple rib fractures, and traumatic pneumothorax. The patient was admitted to the Department of Thoracic Surgery for further management. On hospital day 22, she developed sudden right leg edema with ecchymosis over the distal thigh and was referred to the Department of Vascular Surgery with suspicion of deep vein thrombosis (DVT). The patient reported that ambulation had been initiated 1 week before the onset of right leg swelling.

Duplex ultrasonography of the right lower extremity revealed no evidence of DVT; however, a 1.3×1.4 cm pseudoaneurysm (PSA) was identified above the knee of the right leg (Fig. 1A, 1B). Three-dimensional computed tomography angiography (3D CTA) of the lower extremity was performed the following day to determine the exact origin of the lesion. 3D CTA revealed that the PSA originated from a branch of the superior medial genicular artery (Fig. 1C, 1D). Although ultrasound (US)-guided compression was initially attempted, US-guided thrombin injection was performed, given the small size of the PSA (<2 cm) and its deep location approximately 2.7 cm beneath the skin surface. Specifically, with the patient in the supine position, a 21-gauge, 3-cm needle was inserted under US guidance without local anesthesia (Fig. 2A). Thrombin (500 U, reconstituted in 5 mL distilled water) was slowly injected until complete cessation of the flow within the PSA was confirmed using color Doppler imaging. Ultrasonography revealed thrombosis of the PSA sac with complete occlusion (Fig. 2B). Furthermore, flow within the popliteal and distal arteries was verified to be intact. Follow-up ultrasonography performed 24 hours later showed no residual arterial flow within the PSA (Fig. 2C). At the 52-day outpatient follow-up, complete resolution of the right lower extremity edema and ecchymosis was noted, and no further ultrasonographic evaluation was performed.

Traumatic PSAs of genicular arteries are rare. In nontraumatic settings, PSAs involving the genicular branches have occasionally been reported after total knee arthroplasty or arthroscopic knee procedures [1,2].

In cases of blunt trauma, if delayed lower extremity swelling and ecchymosis develop after the initial injury, it is necessary to carefully consider the possibility of vascular injury. When genicular artery injury is suspected, a diagnostic evaluation can be performed using duplex ultrasonography, computed tomography angiography, or conventional diagnostic angiography [3]. Genicular artery PSAs are commonly managed using minimally invasive techniques. US-guided manual compression or thrombin injection is preferred for small lesions (<2 cm), whereas endovascular embolization (coil or glue) is preferred for larger lesions or symptomatic cases. Open surgical ligation or resection is reserved for complex or refractory cases, particularly following knee surgery [4]. The lack of imaging beyond 24 hours is a limitation, and follow-up imaging is recommended [5].

US-guided thrombin injections may be considered safe, technically simple, and less invasive treatment options for traumatic PSAs, potentially serving as alternatives to endovascular interventions or open surgical repairs.

## Figures and Tables

**Fig. 1.**
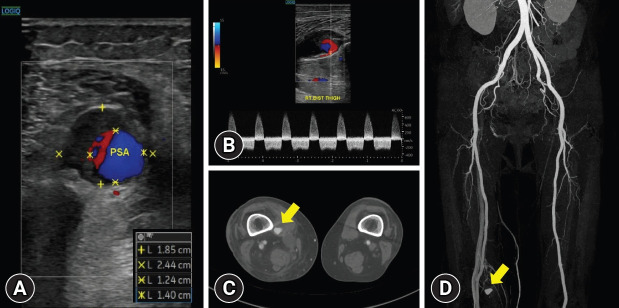
Preprocedural duplex ultrasonography and computed tomography. (A) Swelling is observed in the right lower extremity. A pseudoaneurysm (PSA) is identified in the right distal thigh. (B) Color Doppler imaging showing the “yin–yang sign” within the pseudoaneurysm sac, and a characteristic “to-and-fro” flow pattern is observed at the neck. (C) Axial computed tomography image showing the pseudoaneurysm (arrow). (D) Computed tomography angiography demonstrating the pseudoaneurysm (arrow).

**Fig. 2.**
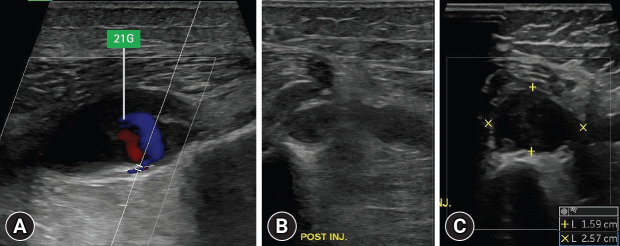
Thrombin injection of a pseudoaneurysm. (A) Schematic illustration. (B) Post-procedural ultrasonographic image. (C) Ultrasonographic image at 24-hour follow-up. Complete occlusion of the pseudoaneurysm is demonstrated immediately after the procedure and at 24-hour follow-up.
